# Travis County Medical Society

**Published:** 1908-01

**Authors:** 


### Society Notes.

Travis County Medical Society.—Annual meeting, Austin, December 18. Paper by Professor Bantel, of the University of Texas: "Sewage; Its Disposal." Officers for 1908: President, J. W. McLaughlin, Sr.; Vice-President, W. A. Harper; Secretary, H. C. Decherd (re-elected). Delegate to State Medical Association (two years), Dr. F. E. Daniel; Alternate, Dr. H. B. Hill.

